# Diagnostic challenges of posterior fossa hemangioblastomas: Refining current radiological classification scheme

**DOI:** 10.1038/s41598-020-63207-0

**Published:** 2020-04-14

**Authors:** Eui Hyun Kim, Ju Hyung Moon, Seok-Gu Kang, Kyu Sung Lee, Jong Hee Chang

**Affiliations:** 1 Department of Neurosurgery, Brain Tumor Center, Yonsei University College of Medicine, Seoul, Republic of Korea; 2 Department of Neurosurgery, Gangnam Severance Hospital, Yonsei University College of Medicine, Seoul, Republic of Korea

**Keywords:** CNS cancer, Surgical oncology

## Abstract

Hemangioblastomas (HBMs) are known to exhibit very typical radiological features and thus classified by well-established radiological classification scheme. We reviewed our series of posterior fossa HBMs in order not only to evaluate the relevance of current classification scheme, but also to possibly refine it. Also, we added descriptions on several cases with unusual radiological magnetic resonance imaging (MRI) findings in which differential diagnosis was challenging. We retrospectively reviewed preoperative MRI of 118 patients with pathologically diagnosed posterior fossa HBMs at our institution between 2002 and 2015. Total 128 tumors were included to this study and classified into four categories based on the presence and nature of cystic components: extratumoral cystic (Type Ce, classical cystic with a mural nodule), intratumoral cystic (Type Ci), mixed cystic (Type Cm), and solid (Type S). The association with von Hippel-Lindau (VHL) disease was also investigated. In 118 patients (65 male and 53 female), 79 (66.9%) had solitary HBMs and 39 (33.1%) were diagnosed with VHL disease. Type Ce with typical radiological findings was the most prevalent type of HBM (63.3%), followed by Type S (21.1%). HBMs with intratumoral cysts were uncommon (Type Ci, 11.7%) and mixed extratumoral and intratumoral cysts (Type Cm) accounted for only 3.9%. No intergroup differences were observed in the proportions of each subtype between the solitary and VHL disease-associated HBMs. The blood loss was much lower in Type Ce than in other subtypes. In Type Cm, radical resection was often challenging as the differentiation between intratumoral and extratumoral cysts was difficult resulting in incomplete resection. Refined radiological classification scheme is more practical because it does not only help surgeons determine whether the cystic wall should be removed or not, but also covers cases with atypical radiological presentations. For solid and extraparenchymal HBMs, differential diagnosis is more difficult as well as very critical as surgical removal is often very challenging.

## Introduction

Hemangioblastomas (HBMs) are highly vascular tumors most commonly occurring in the cerebellum, brain stem, and spinal cord. HBMs occur both sporadically and as an important component of von Hippel-Lindau (VHL) disease^1–3^. VHL disease is an autosomal dominant inherited disease classically associated with neoplasms in multiple organs, and is caused by mutations in the *VHL* gene on chromosome 3p25-p26^1,4^. HBMs present quite typical radiological findings of a strongly contrast-enhanced nodule with a cystic component on magnetic resonance imaging (MRI), which is often pathognomic; however, they also commonly appear as purely solid tumors. HBMs have been traditionally classified into four types based on the proportion of cystic and solid components^5^. Type 1 (simple cyst type, 6%) is characterized by a cyst with clear fluid and smooth walls and without evidence of a mural nodule on angiography or at surgery. Type 2 (macrocystic type, 65%) is the most frequent and characterized by a cyst of variable size with a mural nodule. Type 3 (solid type, 25%) has a solid consistency with blurred limits and marked vascularization. Type 4 (microcystic type, 4%) is solid but contains small cysts a few millimeters in size. Recently, a large-scaled meta-analysis also used similar classification system; solid, cystic, cystic with mural nodule and both solid and cystic^6^, which is basically the same as previous classification scheme. However, even with the aid of well-designed radiological classification system, we have encountered significant number of cases with unusual MRI findings that could not be classified into a specific category, thereby resulting in challenges in preoperative diagnosis. In this study, we reviewed our series of posterior fossa HBMs in order not only to evaluate the relevance of current classification scheme, but also to possibly refine it.

## Methods

We retrospectively reviewed the preoperative MRI features of 118 patients with HBMs in the posterior cranial fossa, who underwent surgery at least once and were pathologically diagnosed as having HBMs at our institution between 2002 and 2015. Informed consent was obtained from the patient and all experiments were performed in accordance with relevant guidelines and regulations. This study was approved by the Institutional Review Board of Severance Hospital, Yonsei University College of Medicine.

### Diagnostic criteria of von Hippel-Lindau disease

Diagnosis of VHL disease was made on the basis of clinical and genetic criteria^4^. In the case of a positive family history of VHL disease, a diagnosis of VHL disease was made by identifying a single central nervous system HBM. Clinical diagnosis of VHL disease in patients with no family history required the presence of at least two tumors: two HBMs or a HBM and a visceral tumor, clear cell renal cell carcinoma, pheochromocytoma, pancreatic endocrine tumor, or endolymphatic sac tumor. The detection of a *VHL* mutation by genetic characterization was regarded as sufficient evidence for diagnosing VHL disease in individuals who did not satisfy the other clinical diagnostic criteria.

### Radiological evaluation

**Figure 1:**
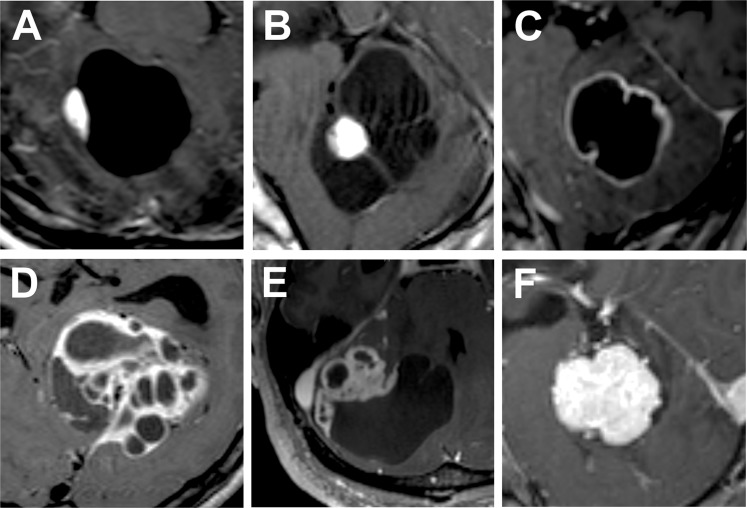
Gadolinium-enhanced T1W images of magnetic resonance imaging of hemangioblastomas (HBMs). Type Ce refers (**A,B**) HBMs of classical radiological features with a main cyst and a mural nodule. Purely cystic HBMs (**C**) used to be an independent subtype, however, they were classified as Type Ci together with HBMs with multiple intratumoral cysts (**D**). Tumors with mixed intratumoral and extratumoral cysts (**E**) were also identified (Type Cm). Solid tumor (F) was the second most common subtype of HBMs.

All patients underwent preoperative and postoperative MRI using a 1.5- or a 3.0-Tesla. All surgically removed tumors were included in the analysis for radiological classification. However, in patients with multiple tumors in VHL disease, most of the small solid tumors with less than 3 mm in longest diameter were excluded from analysis, as they were too small to be characterized for their radiological features. Small asymptomatic tumors with less than 3 mm in longest diameter were mostly solid were primarily detected by regular radiological surveillance. These tumors were usually found in VHL disease patients, which were very small and thus treated using GKS without pathological confirmation. Based on the preoperative MRI features, we classified these tumors based on the traditional classification scheme. After identifying several cases that were not matched to any of categories, we refined the previous classification system as follows: extratumoral cystic (Type Ce, classical cystic with a mural nodule), intratumoral cystic (Type Ci), mixed cystic (Type Cm), and solid (Type S), which are well described in Table 1 and Fig. 1^6^. In our refined classification scheme, both Type 1 (pure cystic) and Type 3 (microcystic) of previous classification system are classified as intratumoral cyst (Type Ci). We identified several HBM tumors which did not truly reside in the brain parenchyma and thus mimicked other type of brain neoplasms. For the comparative analysis, t-test, χ² test, and Fisher’s exact test were used to determine statistical significance with a P-value < 0.05. All statistical analyses were performed using IBM SPSS Statistics (Version 23.0; IBM, Armonk, NY, USA).

**Table 1:** Radiological classification of hemangioblastomas.

Tumor nature	Location of cyst	Type	Previous classification system
Cystic	Extra-tumoral cyst	Type Ce (tumor with non-enhanced cystic wall)	Type 2 (macrocystic)
	Intra-tumoral cyst	Type Ci (tumor with enhanced cystic wall)	Type 1 (pure cystic) Type 3 (microcystic)
	Extra- &d intra-tumoral cysts	Type Cm (mixed)	No category assigned
Solid		Type S (solid)	Type 4 (solid)

### Treatment strategies

For the tumors that presented with profound surrounding edema on preoperative MRI or caused any neurological symptoms, complete surgical resection was performed for immediate decompression irrespective of whether they were solitary or multiple. Cystic walls with contrast enhancement were always surgically removed. For multiple small asymptomatic tumors that could not be reached in the craniotomy for the removal of the main tumors, and for small tumors in the brain stem, gamma knife surgery (GKS, Leksell Gamma Knife; Elekta Instrument, Stockholm, Sweden) was planned. In cases of VHL disease with multiple recurrent tumors, GKS was first considered as the primary treatment option, while a surgical procedure was reserved for future treatment. However, for tumors with a significant cystic component, surgical treatment was always considered as the primary treatment option as their radiosurgical outcomes are relatively unfavorable^7^.

## Results

### General outcomes

In total, 118 patients (65 male and 53 female) were included in this study. Among them, 79 (66.9%) had solitary HBMs and 39 (33.1%) were diagnosed with VHL disease. The mean follow-up duration was 43.8 months (range, 14–80 months). The mean age at initial diagnosis was 45.8 years and 39.9 years for the patients with solitary and VHL disease-associated HBMs, respectively. The sex ratio (Male:Female) was 39:40 among patients with solitary HBMs and 26:13 among those with VHL disease-associated HBMs. Leptomeningeal hemangioblastomatosis developed in two patients after their initial surgical treatments.

## Refined radiological classification scheme

Total 128 tumors in 118 patients were included in the analysis of radiological classification. The result of our radiological classification is summarized in Table 2. Type Ce with typical radiological findings was the most prevalent type of HBM (63.3%), followed by Type S (21.1%). HBMs with intratumoral cysts were uncommon (Type Ci, 11.7%) and mixed extratumoral and intratumoral cysts (Type Cm) accounted for only 3.9% HBMs. No intergroup differences were observed in the proportions of each subtype between the solitary and VHL disease-associated HBMs (P-value = 0.550). In 20 cases of HBMs, tumors presented with multiple significantly large cysts (Fig. 1B,D and E), which was an unusual radiological finding and thus made preoperative diagnosis difficult. Nine of them showed multiple extratumoral cysts, whereas six showed multiple intratumoral cysts. Five cases were categorized into the newly proposed subtype (Type Cm). Multiple cysts were observed both in solitary and VHL disease-associated HBMs.

**Table 2:** Radiological classification of hemangioblastomas (HBMs) in the association with von Hippel-Lindau (VHL) disease.

	Sporadic HBMs	VHL disease-associated HBMs	Total
Type Ce	48 (60.8%)	33 (67.3%)	81 (63.3%)
Type Ci	8 (10.1%)	7 (14.3%)	15 (11.7%)
Type Cm	4 (5.1%)	1 (2.0%)	5 (3.9%)
Type S	19 (24.1%)	8 (16.3%)	27 (21.1%)
Total	79	49	128
P-value			0.550

**Figure 2:**
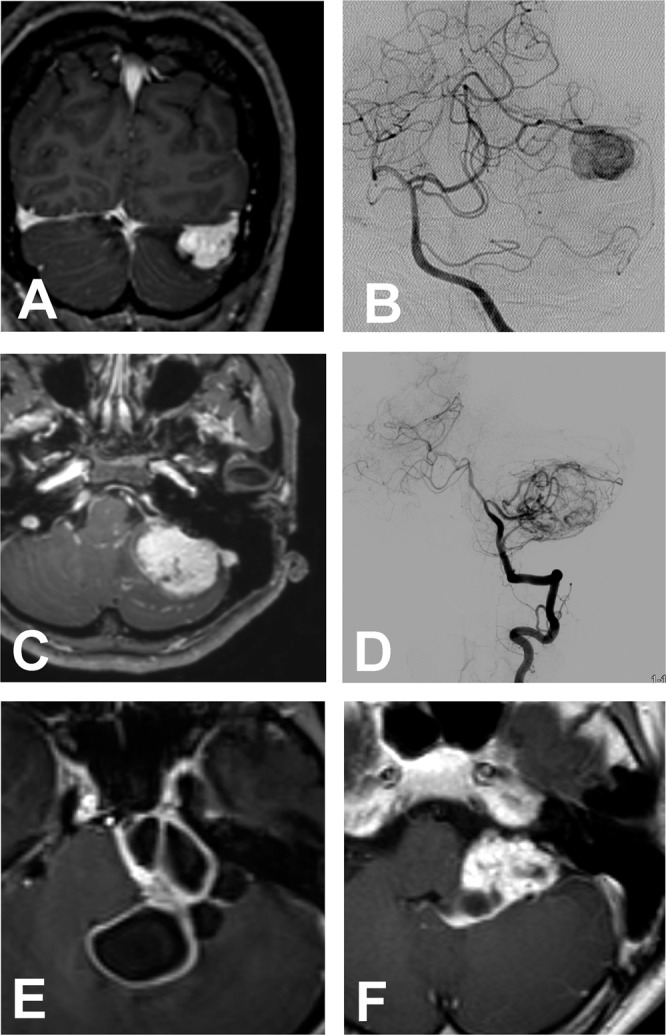
Hemangioblastomas (HBMs) showing unusual radiological findings on Gadolinium-enhanced T1W images of magnetic resonance imaging. Solid hemangioblastomas (Type S) are located on the hemispheric cortex (**A**) and petrous surface (**C**) that mimic meningiomas. Cerebral angiography revealed that these tumors were fed by superior cerebellar artery (**B**) and anterior inferior cerebellar artery (**D**), respectively, which was very helpful in differential diagnosis. A multiple intra-tumoral cystic HBM (Type Ci) is exophyting into a cerebellopontine and cerebellomedullary cistern (**E** and **F**).

We identified 9 extraparenchymal HBMs in which preoperative differential diagnosis was challenging. In 6 patients, they were not entirely located inside brain parenchyma abutting calvaric bone or petrous bone surface, which mimicked meningiomas (Fig. 2). In other 3 patients, the tumors resided in cisternal space mimicking schwannomas or meningiomas. The tumors were purely solid in 7 patients and multiple intratumoral cystic in 2. For these tumors, preoperative cerebral angiography helped in the differential diagnosis.

### Clinical outcomes

There were 2 cases of surgical mortality whose tumors were Type S of HBM. In 111 HBM patients whose operation records were available, patients’ blood loss during operation was compared among each subtypes of HBM. Blood loss was 546.6 ± 538.2 mL, 1350.0 ± 1060.7 mL, 1125.0 ± 809.8 mL and 1208.2 ± 1021.7 mL in Type Ce, Ci, Cm and S, respectively. The blood loss in patients with Type Ce was significantly lower in other patients (P-value = 0.019) whereas the blood loss in Type Ci as much as in solid type of HBMs.

During follow up, we identified total 19 cases of incomplete resection; subtotal resection and recurrence at the resection margin (Table 3). In the comparison with other 109 patients, there was no intergroup difference (P-value = 0.286). Among 3 patients whose tumors resided in cerebellopontine angle mimicking schwannomas or meningiomas, 2 patients underwent incomplete resection; subtotal resection in 1 patient and recurrence at the resection margin in the other.

**Table 3:** Extent resection based on the radiological classification of hemangioblastomas.

	Incomplete resection*	Complete resection	Total
Type Ce	10 (52.6%)	71 (65.1%)	81 (63.3%)
Type Ci	3 (15.8%)	12 (11.0%)	15 (11.7%)
Type Cm	2 (10.5%)	3 (2.8%)	5 (3.9%)
Type S	4 (21.1%)	23 (21.1%)	27 (21.1%)
Total	19	109	128
P-value			0.286

*Incomplete resection includes subtotal resection and recurrence at the resection margin.

### Illustrative case

**Figure 3:**
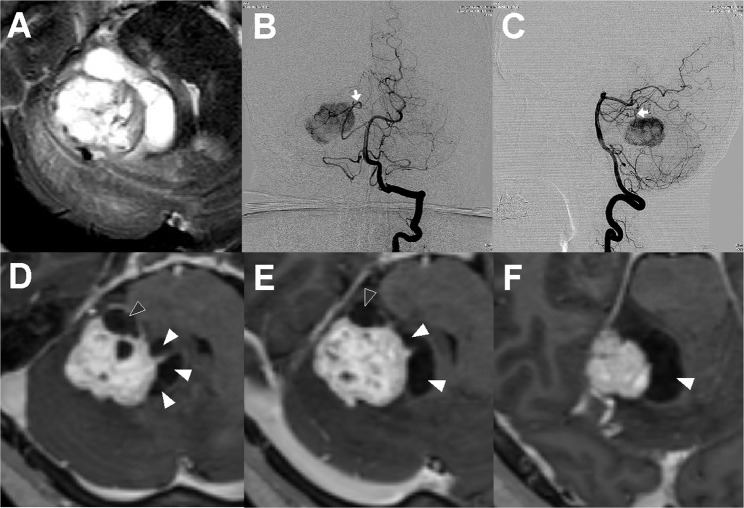
A case of a hemangioblastoma with Type Cm. (**A**) Brain T2-weighted magnetic resonance imaging (MRI) shows multiple cystic components in right cerebellum and middle cerebellar peduncle. Profound peritumoral edema was noted with brain stem compression. (**B,C**) Cerebral angiography revealed tumor feeders come from a superior cerebellar artery located superomedial to the tumor mass. (**D–F**) T1-weighted MRI shows about 3 cm sized strong-enhanced mass with both solid and cystic components. Multiple cystic components were located medial to the main solid mass. Some of the cystic wall showed fairly strong enhancement (black arrow heads) whereas majority of the cysts did not (white arrow heads).

A 53-year-old male patient presented with intermittent dizziness. Brain MRI revealed about 3 cm sized well-enhanced mass with both solid and cystic components in right cerebellum and middle cerebellar peduncle (Fig. 3). Profound peritumoral edema was noted with brain stem compression. Cerebral angiography revealed strong stain on the tumor mass mainly fed by a right superior cerebellar artery (SCA). Apart from small cysts residing inside the main solid tumor, multiple cystic components were located medial to the solid part with direct compression on brain stem and paraventricular structures deep inside. Some of the cystic wall showed fairly strong enhancement whereas majority of the cysts did not. Before surgery, this tumor was classified as Type Cm and the differentiation of the intratumoral and extratumoral cysts was considered critical. During operation, the tumor was accessed with lateral suboccipital approach and tumor feeders from SCA adjacent to extratumoral cysts were safely identified and coagulated. Intratumoral cyst which was located the most caudal and ventral side of the tumor was carefully dissected from brain stem and removed together with the main tumor mass.

## Discussion

Hemangioblastomas are the tumors which show relatively typical radiological features. Cystic tumors with a mural nodule located in posterior cranial fossa is a typical radiological feature of HBMs and often pathognomic by itself^8^. Traditionally, HBMs have been classified into four categories according to their radiological presentations: simple cyst type, macrocystic type, solid type, and microcystic type. This classification system is based on the presence of cystic components and the relative size of the cysts in comparison with the size of entire tumors. After encountering a large number of cases of HBMs, we identified several cases wherein the traditional classification system was insufficient in categorizing the tumors. In order to propose more comprehensive classification scheme to classify these tumors with unusual radiological presentations, we aimed to refine current available radiological classification system. We described the radiological features of each HBM, focusing on the location of the cystic components, i.e., whether the cyst was located inside or outside the tumor. The difference in radiological features between macrocystic and microcystic subtypes proposed in the traditional classification scheme is very apparent, however, they are essentially HBMs with intratumoral cysts; the only difference is the proportion of cyst and solid components. We believe our classification system can be more practical as it is rather intuitive to determine whether the cystic wall should be removed. Whereas cystic wall need not be removed when the cyst is located outside a tumor (extratumoral cyst), the presence of contrast enhancement on the cystic wall (intratumoral cyst) always necessitates the complete removal of the cystic wall together with a mural nodule. It is also noteworthy that some HBMs show simultaneous intratumoral and extratumoral cysts (Type Cm) (Fig. 1E). In these cases, the surgeons should carefully differentiate between the cystic walls they should remove and those they may leave intact. In these cases, intraoperative fluorescence may help the differentiation although further clinical validation is necessary^9–11^. In our refined classification system, there was no intergroup differences in the proportions of each subtype between the solitary and VHL disease-associated HBMs, which was consistent with the result shown in the recent large-scaled meta-analysis^6^.

**Figure 4:**
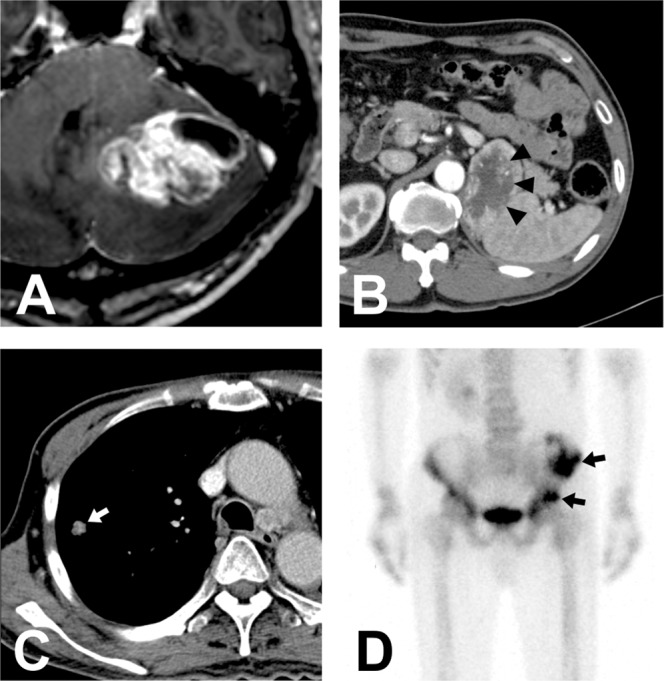
Differentiation between a hemangioblastoma and renal cell carcinoma (RCC). (**A**) A 52-year-old male patient presented a mainly solid tumor with cystic component. The patient has a family history of von Hippel-Lindau disease. (**B**) Abdominal computed tomography revealed cancerous lesion in left kidney (black arrow heads). Moreover, metastasis to lung (**C**), white arrow) and pelvic bone (**D**), black arrows) were identified. Histopathological evaluation for surgically removed tumor also revealed findings consistent with RCC.

Majority of hemangioblastomas arise in the posterior cranial fossa. The presence of cyst either inside or outside tumor mass is always a strong clue to make a diagnosis of hemangioblastomas. When the tumors are multiple, it also increases the possibility of hemangioblastomas associated with VHL disease as well as metastatic tumors. However, when tumor is solitary and especially solid without family history of VHL disease, the differential diagnosis is often challenging as they could be misdiagnosed as other type of brain tumors. For example, the differential diagnosis between purely solid HBMs and metastatic brain tumors is always critical as their radiological features are often similar. Various MRI sequences may help in the differential diagnosis^12–14^. In particular, considering renal cell carcinoma is one of the common clinical manifestations of VHL disease^1,4^, the possibility of metastasis from kidney cancer should always be considered (Fig. 4).

Surgical removal of solid HBMs is often very difficult because the tumor is always surrounded by vigorous arterial blood flow and torturous engorged veins. Compared with cystic HBMs in which surgeons take advantage of the aspiration of the cystic component, surgical maneuverability more limited in a narrow surgical working space with profound cerebral edema. In our study, the fact that blood loss during operation in Type Ce is much lower is in good agreement. The blood loss in Type Ci was as much as in Type S and we believe this is because most of cysts in Type Ci were generally too small. When solid tumors are very large and deep-seated, surgeons should be very careful and well prepared for big challenges during surgery, which consequently makes preoperative radiological diagnosis even more important. Type Cm is a newly proposed subclass comprising only 3.9% of our cases. In this rare type of HBM, the differentiation between intratumoral and extratumoral cysts is always critical during resection of the extremely vascularized tumors as described in Case Illustration (Fig. 3). Indeed, we have experienced 2 cases (10.5%) of Type Cm which led to incomplete surgical resection; subtotal resection in 1 case and recurrence at tumor resection margin in the other (Table 3). Although we failed to show statistical significance because of small number of patients in Type Cm, we believe radical resection is often challenging in Type Cm.

Although MRIs revealed tumors inside cerebellum and brain stem parenchyma in majority of our cases, we experience 9 cases of extraparenchymal tumors, in which tumors were located in a cisternal space or outside cortex abutting calvaric bone and skull base mimicking meningiomas (Fig. 2A–D). Thin-layered pial membrane was identified during surgery in most cases, however, some HBMs were not covered by the pial membrane at all, which made the differential diagnosis difficult even during surgery. In 6 cases of cortical presentation, the tumors lacked dural tail sign, which helped differential diagnosis. Preoperative cerebral angiography was most helpful in the differential diagnosis as meningiomas are mainly fed by external carotid artery and its branches whereas HBMs are supplied from a vertebrobasilar system^15^. Also, presence of large serpentine veins and feeding arteries around tumors on MRI is also an important sign suggestive of HBMs. The most challenging cases in our experience was cisternal type of HBMs (Fig. 2E,F). We experienced total 3 cases of cisternal extraparenchymal HBMs; 1 solid tumor and 2 multiple intratumoral cystic tumors. They were seated deep in the cerebellopontine and cerebellomedullary cistern mimicking tentorial or petrous apex meningiomas and a schwannoma. As internal debulking is always necessary for safe resection of the tumor in this location, we had to cope with brisk bleeding during entire procedure.

One of the limitations in this study is that we excluded small asymptomatic tumors that were followed up without surgery from our analysis. All of these tumors seemed to be solid on MRI, however, we thought it was too early to determine the presence and location of the cystic component. However, as all of these tumors were observed in the patients with VHL disease, this might influence the proportion of each subtypes of HBMs in a VHL disease group. Second, we did not perform biopsy from the extratumoral cyst in Type Ce and Type Cm whereas pathological examination was always performed to check for the presence of tumor cells in the surgically removed cystic wall in Type Ci. Therefore, the differentiation between intratumoral and extratumoral cysts only depends on preoperative imaging and intraoperative finding, and is not supported by histopathological confirmation.

## Conclusions

Radiological features of HBMs are often typical thus well classified based on the presence and location of the cyst. However, it is also important to recognize cases with unusual radiological presentations. We refined the traditional radiological classification scheme covering atypical HBMs which have not been categorized in the previous classification system. Our suggestion is also more practical as it helps surgeons determine whether the cystic wall should be removed or not, but also covers cases with atypical radiological presentations. For solid HBMs, differentiation from metastatic brain tumors is always critical. Especially in VHL disease, surgeon should be aware of a possibility of metastasis from renal cell carcinoma. Preoperative presumption is always critical in extraparenchymal HBMs as surgical resection.is the most challenging.
